# Predictors and Prognostic Implications of Prolonged Operative Time in Rectal Cancer Surgery: An Analysis Adjusted for Surgeon and Surgical Approach

**DOI:** 10.3390/jcm15218473

**Published:** 2026-10-30

**Authors:** Dan Assaf, Nadav Elbaz, Yasmin Anderson, Ofir Gruper, Lior Bear, Eyal Mor, Niv Pencovich, Gali Westrich, Lior Segev

**Affiliations:** 1 Division of Surgery, The Chaim Sheba Medical Center, Ramat Gan 52621, Israel; 2 Gray Faculty of Medicine, Tel-Aviv University, Tel-Aviv 69978, Israel

**Keywords:** rectal cancer, operative time, outcomes

## Abstract

**Background/objectives:** To analyze risk factors for prolonged operative time (OT) in patients undergoing rectal cancer surgery, and to explore the effects of prolonged OT on immediate surgical outcomes and long-term oncological outcomes. **Methods:** A single-center retrospective review of all patients who underwent an elective rectal resection for primary rectal adenocarcinoma between 2010 and 2020. OT was standardized according to surgeon-specific and surgical approach-specific median values. Subsequently, the cohort was divided according to surgery duration: The Short OT group (244 patients; median OT 201 min, range 46–600) and the Long OT group (250 patients; median OT 340 min, range 145–960). **Results:** Multivariate analysis identified independent predictors for prolonged surgery: higher BMI (AOR 1.07, *p* = 0.002) and distance from anal verge (AOR 0.89, *p* < 0.001). Intraoperative complications occurred more frequently in the Long OT group (21.6% vs. 10.2%, *p* < 0.001), as did overall post-operative morbidity (73.2% vs. 60.7%, *p* = 0.003). The 5-year OS rates were comparable between groups (88.8% vs. 90.8%, *p* = 0.14); however, 5-year RFS was significantly worse in the Long OT group (65.5% vs. 78.2%, *p* = 0.004). Multivariable Cox regression using surgeon–approach standardized time quartiles showed reduced recurrence-free survival per one-quartile duration increase (HR 1.37, 95% CI 1.12–1.69, *p* = 0.002). **Conclusions:** Higher BMI and lower tumor location were found to be independent preoperative predictors of prolonged rectal cancer surgery. Prolonged operative time was associated with higher perioperative morbidity and poorer long-term recurrence-free survival.

## 1. Introduction

Operation time (OT) is considered one of the key elements influencing surgical outcomes [1]. Prolonged OT has been associated with increased post-operative morbidity and mortality in colorectal surgery as well, including higher rates of surgical site infections, anastomotic leaks, blood transfusion, reoperation, prolonged hospital stay, and readmissions [2,3,4,5]. However, some other studies have shown contrasting results [6,7,8]. Operative time is a surrogate for surgical complexity, and it is influenced by multiple factors, including patient factors such as anatomy and body habitus, disease factors such as tumor size, tumor location, and neoadjuvant radiation, surgeon experience, and operative factors such as operative approach, operative findings, and complexity. Better defining the possible correlation between OT and surgical outcomes could potentially allow the development of a marker for surgical complexity, which could be used for risk stratification and predicting adverse events. Minimally invasive colorectal surgery (laparoscopic or robotic) is technically challenging and requires advanced training, and one of its main drawbacks is that it takes longer to perform than open procedures [9]. It is not clear if the proven benefits of minimally invasive surgery in immediate surgical outcomes are also maintained in long-duration colorectal procedures.

Although there have been studies concerning the association between OT and post-operative outcomes in colorectal surgery, there have been no consistent conclusions. Most of the previous studies have either combined colon and rectal surgeries together or focused on colon resections alone [10,11]. Currently, existing research specifically investigating the correlation between operative duration and rectal cancer surgical outcomes remains scarce. Additionally, these studies have focused solely on the association between prolonged OT and immediate surgical outcomes, specifically in minimally invasive approaches [12]. To the best of our knowledge, no study has yet examined the effect of prolonged OT specifically on rectal cancer surgery outcomes, including long-term oncological outcomes.

Therefore, the aim of this study is to analyze risk factors for prolonged OT in patients undergoing rectal cancer surgery and to explore the effects of prolonged OT on post-operative surgical outcomes and long-term oncological outcomes in these patients.

## 2. Materials and Methods

Conducted as a single-center investigation at an Israeli tertiary referral hospital, this study received institutional review board approval (reference 9618-22-SMC). By searching electronic medical records, researchers identified patients who underwent elective rectal cancer resections between 2010 and 2020. Standardized coding was utilized to retrieve specific surgical interventions, such as abdominoperineal resections (APRs), anterior resections, and proctectomies incorporating coloanal anastomoses. The final cohort was restricted to individuals with primary rectal adenocarcinoma diagnosed at clinical stages I through III. Cases involving palliative care, metastatic disease, non-elective or emergency admissions, or missing operative duration records (totaling 32 patients) were omitted from the analysis. (Table S1). This dataset shares an overlapping institutional database with our prior work evaluating BMI categories in rectal cancer [13]. However, the present analysis is distinct, focusing on surgeon- and approach-standardized operative time, its preoperative predictors, and long-term oncological outcomes. For patients who received neoadjuvant therapy, restaging was systematically performed 6–8 weeks post-treatment completion using pelvic MRI, rigid or flexible endosigmoidoscopy, and clinical examination prior to surgery.

Post-operative complications were classified according to the Clavien–Dindo system. Overall complications were defined as any Grade I–V event. Severe complications were defined as Clavien–Dindo Grade ≥ III (requiring surgical, endoscopic, or radiological re-intervention, or intensive care unit management). For patients experiencing multiple concurrent post-operative events, the highest overall Clavien–Dindo grade was assigned to represent the patient’s severe morbidity.

To account for inherent variability in operative duration—specifically surgeon’s experience and the distinction between open and minimally invasive approaches—a median-based standardization was employed. Operations were first stratified by the primary surgeon and the specific surgical approach (open vs. minimally invasive). For each surgeon–approach combination, a specific median operative time was calculated. Individual cases were then categorized based on their position relative to these medians. Standardized duration was modeled both as a binary in relation to the standardized median and ordinally across standardized quartiles. The cohort was subsequently dichotomized into the following two comparative groups: Short OT group: Cases falling below the standardized median (*n* = 244); Long OT group: Cases falling above or equal to the standardized median (*n* = 250).

Data were gathered into a REDCap database. Data analysis was performed using SPSS version 30 software (Armonk, NY, USA: IBM Corp). All statistical analyses were conducted with a significance level of α = 0.05. Continuous variables (age, BMI, preoperative albumin level, preoperative hemoglobin level, distance from the anal verge, pathological distance from the resection margin, and pathological tumor size) were assessed for distribution using skewness and kurtosis values, with absolute values below 2.0 considered approximately normal. Normally distributed continuous variables are presented as means and standard deviations and were compared between groups using the Student’s *t*-test. All other variables were treated as categorical and are presented as frequencies and percentages. Categorical variables were compared using the χ^2^ test or Fisher’s exact test, as appropriate.

Multivariable statistical modeling was conducted using two distinct analytical approaches. First, multivariable binary logistic regression was performed to identify independent predictors of prolonged operative time (Long OT). Adjustment was strictly restricted to baseline preoperative predictors known prior to surgery (age, sex, BMI, tumor distance from the anal verge, and preoperative chemoradiation); intraoperative characteristics, complications, and final pathological features were excluded. Second, multivariable Cox proportional hazards regression was used to evaluate recurrence-free survival (RFS), incorporating baseline, operative, and pathological covariates. Recurrence-free survival was defined as the duration from primary surgery to the date of first radiologically, clinically, or pathologically documented disease recurrence. Patients who died without documented recurrence were censored at the date of last recurrence-free follow-up (non-cancer deaths were not counted as RFS events). Overall survival (OS) was defined as time from surgery to death from any cause (50 total death events; *n* = 20 in Short OT vs. *n* = 30 in Long OT). Candidate variables with *p* < 0.10 in univariate screening or established clinical relevance were included. For the RFS model (*N* = 489, 122 recurrence events), events-per-variable thresholds met standard guidelines (>10 EPV). Multicollinearity among pathological variables (T stage and overall stage) was assessed using Variance Inflation Factor (VIF) diagnostics, with values < 2.5 indicating no significant collinearity. Complete case analysis was utilized (<2% missing data across variables). Complete-case analysis yielded *n* = 493 for the logistic regression model (1 missing BMI value) and *n* = 489 for the Cox proportional hazards models (excluding 4 missing pathological staging values and 1 missing BMI value).

To evaluate factors linked to surgical duration, univariate assessments utilized binary logistic regression, while multivariate evaluations applied a binary logistic regression model employing a forward likelihood ratio approach. Multivariable binary logistic regression was performed to identify independent preoperative predictors of prolonged operative time, with adjustment restricted strictly to baseline factors known prior to surgery (age, sex, BMI, tumor distance from the anal verge, and preoperative chemoradiation). Separately, multivariable Cox proportional hazards regression was used to evaluate recurrence-free survival (RFS), incorporating relevant baseline, operative, and pathological covariates. For survival evaluations, Kaplan–Meier curves coupled with log-rank significance testing were applied. Multivariate survival analyses evaluated recurrence-free survival through Cox regression models via the forward likelihood ratio method, expressing hazard ratios relative to every single-quartile rise in standardized surgery length. Similar to the previous model, multivariable selection relied on clinical relevance and published literature, restricted specifically to baseline preoperative confounders.

## 3. Results

Overall, 494 patients met the inclusion criteria and were included in the analysis. The Short OT (*n* = 244) had a median surgery duration of 201 min (range 46–600), and the Long OT (*n* = 250) had a median surgery duration of 340 min (range 145–960), *p* < 0.001.

### 3.1. Patient Characteristics

The two groups were similar in age, with a mean age of 63.1 years (range, 31–88) in the Short OT and 63.7 years (range, 31–86) in the Long OT (*p* = 0.59). However, the Long OT included a significantly higher proportion of male patients (61.2% vs. 50.8%, *p* = 0.02), and mean BMI was significantly higher in the Long OT (27.23 versus 26.06, *p* = 0.003). Otherwise, the two groups were comparable in terms of baseline characteristics and comorbidities (Table 1).

### 3.2. Disease Presentation

The proportion of symptomatic patients was similar between groups (77.9% vs. 82.4%, *p* = 0.21), with no significant differences in presenting symptom prevalence (all *p* > 0.05).

Preoperative tumor markers were comparable between the groups (CEA—*p* = 0.29, CA 19-9, *p* = 0.9).

Tumors in the Long OT group were located significantly lower in the rectum compared to the Short OT (*p* < 0.001), with higher proportions of mid-rectal (40% vs. 24.2%) and lower rectal tumors (15.2% vs. 7.8%), whereas upper rectal tumors were more common in the Short OT (68% vs. 44.8%). Neoadjuvant therapy was utilized more frequently among the Long OT group, specifically, long-course neoadjuvant radiotherapy (52% vs. 35.2%, *p* < 0.001) and total neoadjuvant therapy (5.6% vs. 2%, *p* = 0.04), while short-course radiotherapy rates were similar (*p* = 0.76).

Clinical stage distribution differed slightly between groups (*p* = 0.049), with a higher proportion of stage III disease in the long OT (52% vs. 46.3%). Nodal clinical staging was also more advanced in the Long OT (N2 disease: 16% vs. 11.5%, *p* = 0.033), while T stage distribution showed a trend toward more advanced tumors in the Long OT that did not reach statistical significance (*p* = 0.052). (Table 2).

### 3.3. Operative and Perioperative Outcomes

The distribution of surgical approaches was similar between the groups, with no significant differences in the rates of open, laparoscopic, or robotic surgery (*p* = 0.67). Among attempted minimally invasive resections (*n* = 364; 181 Short OT vs. 183 Long OT), the overall conversion rate was 19.0% (69/364). There was a trend towards more conversions from minimally invasive procedures (lap/robot) to an open approach among the Long OT (14.9% conversion rate versus 22.9%, *p* = 0.051).

Surgical procedures differed significantly between the groups, with low anterior resection performed more frequently in the Long OT (67.6% vs. 51.2%), and abdominoperineal resection, as well (8.8% vs. 4.1%, *p* = 0.03).

Extended resections were significantly more common in the Long OT (21.6% vs. 9.8%, *p* < 0.001), including higher rates of additional anastomoses, bilateral salpingo-oophorectomy, and hysterectomy. Intraoperative complications occurred more frequently in the Long OT (21.6% vs. 10.2%, *p* < 0.001), particularly enterotomy (6.4% vs. 2%, *p* = 0.02). Diversion stoma creation was significantly more common in the Long OT (77.2% vs. 45.9%, *p* < 0.001) (Table 3).

Regarding overall morbidity, 73.2% of patients in the Long OT group experienced a complication compared to 60.7% in the Short OT group (*p* = 0.003). When evaluating clinically significant severe morbidity (Clavien–Dindo grade ≥ III), there was a trend toward a higher incidence in the Long OT group compared to the Short OT group (19.6% vs. 13.1%, *p* = 0.052). Individual complication rates, including intra-abdominal abscess (10% vs. 4.5%, *p* = 0.02) and post-operative bleeding/blood transfusion (17.6% vs. 10.2%, *p* = 0.02), were more common in the long OT. Rates of surgical site infection, ileus or small bowel obstruction, anastomotic leak, pneumonia, urinary tract infection, thromboembolic events, and wound complications were similar between the groups (all *p* > 0.05). The median Clavien–Dindo score [14] was higher in the Long OT (*p* < 0.001).

The overall median length of stay (LOS) was 8 days (range: 3–98 days), with a median of 8 days in both the Short OT group (range: 3–47 days) and Long OT group (range: 4–98 days) (*p* < 0.001). The most frequently occurring length of stay (mode) was 7 in both groups. There were no significant differences in 30-day readmission (18.4% vs. 17.2%, *p* = 0.73) or 30-day mortality rates (1.6% vs. 0.8%, *p* = 0.69).

### 3.4. Pathological Outcomes

The two groups did not have many significant differences in their pathological characteristics. The rate of complete pathological response was similar (26% vs. 18.9% in the Short and Long OT groups, respectively, *p* = 0.19). There were no significant differences in pathological staging (*p* = 0.26), pathological T staging (*p* = 0.44), or pathological N staging (*p* = 0.93). Rates of lymphovascular invasion (LVI) (7.8% vs. 7.6%, *p* = 0.94) and perineural invasion (7.4% vs. 8.4%, *p* = 0.67) were also comparable. However, the Short OT group had a significantly larger mean pathological tumor size (3.25 cm vs. 2.88 cm, *p* = 0.025) and a longer mean distance from the distal margin (3.06 cm vs. 2.29 cm, *p* = 0.008). The two groups were similar also in the median number of harvested lymph nodes (14 in both groups) and in the prevalence of positive resection margins (Table 4).

### 3.5. Survival and Disease Recurrence

**Figure 1:**
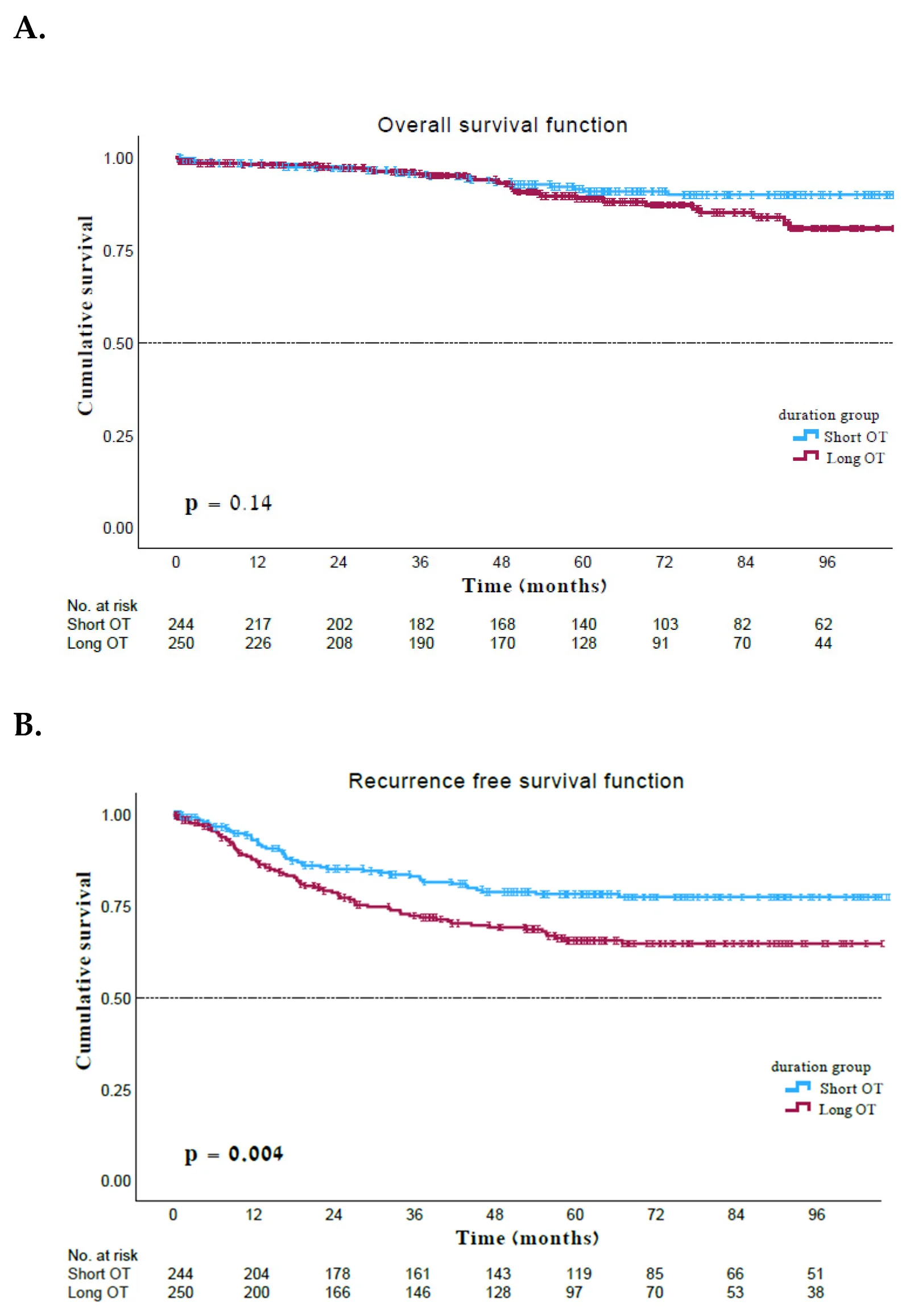
Overall survival (**A**) and recurrence-free survival (**B**) Kaplan–Meier curves stratified by the study groups.

Following a median follow-up of 62.63 months (range: 1–179 months; Short OT group: 63.3 months; Long OT group: 60.9 months), the analysis revealed that longer surgical duration was significantly associated with worse recurrence-free survival (RFS) (*p* = 0.004), though it did not impact overall survival (OS) (*p* = 0.14). While the estimated median RFS was not reached in either cohort, the five-year RFS rate was significantly lower in the Long OT group compared to the Short OT group (65.5% vs. 78.2%, respectively; *p* = 0.004). Similarly, the estimated median OS was not reached for either group, and five-year OS rates remained comparable between the two groups (88.8% vs. 90.8%, *p* = 0.14) (Figure 1).

### 3.6. Univariate and Multivariate Analysis of Factors Associated with Recurrence-Free Survival

In a univariate analysis, several factors were significantly associated with recurrence-free survival (RFS), including shorter tumor distance from the anal verge (AV) (*p* = 0.023), elevated preoperative CEA levels (*p* < 0.001), longer surgery duration (*p* = 0.002), and pathological mucinous features (*p* = 0.024). Pathological indicators such as T stage, overall pathological stage, and margin involvement (distal/radial) also demonstrated strong significance (all *p* < 0.001). In the multivariate model, margin involvement (distal/radial) was the strongest independent predictor of worse RFS (HR 3.4, 95% CI 1.54–7.51, *p* = 0.002). Pathological T stage (HR 1.82, 95% CI 1.28–2.58, *p* < 0.001), surgeon–approach standardized surgery duration quartile (HR 1.37, 95% CI 1.12–1.69, *p* = 0.002 per one-quartile increase), pathological stage (HR 1.19, 95% CI 1.01–1.42, *p* = 0.04), and distance from the AV (HR 0.95, 95% CI 0.91–0.99, *p* = 0.035) also remained significant risk factors for disease recurrence (Table 5). To test the robustness of our findings, a sensitivity analysis was performed excluding overall pathological stage from the multivariable Cox regression model. This demonstrated that the surgeon–approach standardized operative time quartile remained a significant independent predictor of reduced recurrence-free survival (HR = 1.39, 95% CI 1.14–1.71, *p* = 0.002).

### 3.7. Predictors for Prolonged Surgery Time

Several factors were identified as significant predictors of longer surgery duration in univariate and multivariate analyses. In univariate analysis, male sex (*p* = 0.02), higher BMI (*p* = 0.005), shorter distance from the anal verge (AV) (*p* < 0.001), and preoperative long-course chemoradiation (*p* < 0.001) were associated with longer surgery duration. In the multivariable model restricted to preoperative factors, higher BMI (AOR 1.07, 95% CI 1.02–1.11, *p* = 0.002) and shorter distance from the AV (AOR 0.89, 95% CI 0.85–0.93, *p* < 0.001) remained independent predictors of prolonged operative duration. Conversely, factors such as age (*p* = 0.53), preoperative long-course chemoradiation (*p* = 0.35), and male sex (*p* = 0.059) did not reach statistically significant independence in the final adjusted model (Table 6).

## 4. Discussion

The previous literature analyzing the impact of operative time on outcomes in colorectal surgery has either focused solely on colon resections [15], or combined colon and rectal resections together [16], or analyzed open and minimally invasive procedures together [15,17], and the vast majority of those studies have related only to immediate surgical outcomes without analyzing the effect of prolonged surgery on long-term outcomes [15,16,18]. In addition, these studies have divided their cohorts arbitrarily according to either the overall median OT or OT quartiles, therefore not accounting for the inherent variability in operative duration—specifically surgeon’s experience and the distinction between open and minimally invasive approaches. Our current study is unique in focusing specifically on rectal cancer surgery, analyzing the impact of prolonged surgery on both immediate and long-term outcomes, and, most importantly, we standardized operative time according to surgeon-specific and surgical approach-specific median values.

We found higher BMI and shorter distance from the AV to be independent predictors of prolonged rectal cancer surgery in multivariable analysis restricted to preoperative factors. While male sex and preoperative long-course chemoradiation were significantly associated with prolonged operative duration in univariate analysis, they did not retain independent statistical significance in the final adjusted model (*p* = 0.059 and *p* = 0.35, respectively). It should be noted that male sex itself is an imperfect surrogate for complex anatomical parameters, such as narrow interspinous distances, reduced pelvic depth, and increased mesorectal bulk, which are the true drivers of technical difficulty. Although our study lacks MRI-based pelvimetry parameters, our findings overlap with previous publications, and the factors that make rectal cancer difficult are consistent across most prior publications [19,20,21,22]. In a recent cohort of 382 robotic low anterior resections, Yuval et al. showed that male gender, high BMI, neoadjuvant radiotherapy, low tumor height, and large mesorectal area were all independent predictors of longer OT [23]. Similarly, in another series of 166 consecutive robotic TME multivariate analyses revealed that BMI, tumor distance from the anal verge, and the posterior rectal mesenteric thickness were independent predictors of surgical duration [12]. Furthermore, comparative studies evaluating robotic versus laparoscopic TME in technically challenging male pelvises demonstrate that prolonged operative duration does not denote inferior dissection quality or compromised oncological control; rather, robotic TME in male patients often requires longer operative time yet yields comparable or superior mesorectal specimen completeness and local control compared to laparoscopy [24,25]. While these comparative studies are subject to retrospective selection bias and confounding by approach, they reinforce the concept that operative duration reflects anatomical complexity rather than poor surgical quality.

Elevated body mass index is associated with higher mesorectal fat area and higher visceral fat volume [26], limiting the exposure of the operative field, limiting the exposure of blood vessels and other critical anatomical landmarks in the pelvis, and restricting the already restricted pelvic operative space, all together increasing the degree of surgical difficulty and eventually leading to longer operating times [13,27].

Additionally, while a higher clinical N-stage was observed in the Long OT group, advanced nodal involvement alone does not inherently prolong operative duration. Although our database did not systematically record lateral pelvic lymph node dissections, it is reasonable to assume that advanced nodal disease contributed to greater technical difficulty during TME, required more meticulous pelvic tissue clearance, and correlated with the higher rate of extended resections observed in this cohort (21.6% vs. 9.8%, *p* < 0.001).

We found that prolonged OT was associated with increased operative and post-operative morbidity. These results agree with multiple prior studies that have identified a strong correlation between longer operative times and post-operative adverse events following colorectal surgery. In a cohort of 113,615 colectomy patients from the NSQIP database, it was found that patients with long procedures had 34% more SSI, 65% more organ space infections, 69% more abdominal dehiscence, 45% more thrombotic events, 40% more returns to the operating room, and 36% more prolonged length of stay (*p* < 0.05 for all analyses) [16]. Similarly, Guidolin et al. analyzed 26,380 segmental colectomies from the NSQIP datasets and showed that increased OT was linearly associated with any complication (OR = 1.003, 95% CI 1.003–1.003, *p* < 0.0001) in the overall cohort, as was length of stay (*p* < 0.0001), and that each additional 30 min increases the odds of complication by 10% [15]. It is important to emphasize that the observed relationship between prolonged operative duration and increased post-operative morbidity represents an association rather than direct causality. Intraoperative complications and technical challenges (such as difficult pelvic exposure in narrow male pelvises or low rectal tumors) inherently prolong operative duration while independently increasing post-operative morbidity. Thus, operative time largely serves as an integrative surrogate marker for overall surgical complexity, anatomical difficulty, and intraoperative events.

The impact of operation duration on surgical outcomes is debatable. There is evidence that prolonged operations result in greater surgical trauma, increased risk of thromboembolism, prolonged exposure to pathogens, and suppression of the patient’s immune system [28]. Specifically in MIS procedures, longer OT results in prolonged exposure to CO2 pneumoperitoneum, which may negatively impact cardiovascular and respiratory function by reducing venous return and cardiac output and increasing peripheral vascular and pulmonary resistance [29].

Despite having similar surgical oncology quality metrics (lymph node harvesting and surgical margin clearance), we found prolonged surgery duration to be an independent risk factor for disease recurrence. One possible explanation is the more advanced clinical staging among the Long OT group. However, consequently, they also received more neoadjuvant therapy and eventually presented similar pathological results. Similar to our results, in an analysis of 6224 colorectal surgeries, it was shown that shorter operation duration was associated with higher OS and RFS in CRC patients undergoing colorectal resection. The 5-year OS was 82.0% in the short operative duration group compared with 76.7% in the long operative duration group, and the 5-year RFS was 76.2% in the Short OT group versus 68.7% in the Long OT group (*p* < 0.001) [17]. It is crucial to emphasize that the observed independent association between prolonged operative duration and reduced recurrence-free survival (HR = 1.37, 95% CI 1.12–1.69, *p* = 0.002) represents an epidemiological correlation rather than direct causality. Artificially hastening or shortening operative time would not alter underlying tumor biology. Although the exact mechanisms underlying this possible association between prolonged operation and poor long-term prognosis are not fully understood, we speculate that surgical complexity is a surrogate marker for more biologically aggressive tumors that have responded less to neoadjuvant therapy, eventually leading to more difficult and prolonged surgery. Another plausible link is that the immediate increased morbidity associated with prolonged operations leads to a delayed and slow recovery, which might postpone or even prevent patients from getting adjuvant therapy.

Although it warrants further study, this association between prolonged OT in rectal cancer surgery and poorer long-term prognosis should not be ignored. It was already shown that the surgical approach and surgeons’ experience and qualifications are two important modifiable factors affecting operation duration [17]. Therefore, while artificially hastening surgery cannot alter tumor biology, striving for efficient operative flow, minimizing avoidable delays, and refining surgical technique remain essential for reducing perioperative morbidity and avoiding delayed recovery [6], setting up a minimum number of supervised rectal cancer procedures before approving completely independent rectal cancer surgeons, and strengthening the cooperation of all different teams in the operating room. In addition, we should keep in mind that prolonging rectal cancer surgery unnecessarily might lead to a significant negative impact on the patient’s outcomes. Therefore, despite those operations being fundamentally long, surgeons should strive for efficient operative flow, minimize delays, and find the right balance between our important teaching commitment and our obligation to the patient.

Our study has several limitations. First, its retrospective design may have caused potential selection bias. Additionally, the study was conducted at a single specialized cancer center, which may limit its generalizability. Additional limitations are based on the heterogeneous patient risk factors, surgeon expertise, and lack of randomization. Our dataset spans a 10-year period, during which treatment protocols for rectal cancer have changed. Specifically, the TNT protocol was implemented just toward the end of our study; therefore, our results may not fully represent current protocols. Furthermore, because Clavien–Dindo severity was recorded as an overall grade per patient rather than mapped to individual line items, we could not isolate which specific complication drove Grade ≥ III morbidity in patients with multiple concurrent events. Additionally, the comparison of post-operative complications between operative duration groups was unadjusted; because intraoperative complications and surgical complexity factors (such as lower tumor height and extended resections) can both prolong operative time and directly increase post-operative risk, a bidirectional relationship exists that precludes causal inference. Finally, notwithstanding thorough data acquisition, our study database did not capture details regarding estimated blood loss, the thoroughness of mesorectal excision, or lateral pelvic lymph node dissections—all of which serve as key indicators for technical complexity and oncological adequacy.

## 5. Conclusions

We identified higher BMI and shorter tumor distance from the anal verge to be independent preoperative predictors of prolonged rectal cancer surgery. It should be acknowledged that such a prolonged operation is associated with higher perioperative morbidity and possibly poorer long-term oncological outcomes.

## Figures and Tables

**Table 1:** Patient demographics and characteristics.

Variable	All Cohort (*n* = 494)	Short OT (*n* = 244)	Long OT (*n* = 250)	*p* Value
Age in years, mean (range)	63.52 (31–88)	63.09 (31–88)	63.69 (31–86)	0.59
Gender				**0.02**
Male, *n* (%)	277 (56.1%)	124 (50.8%)	153 (61.2%)	
Female, *n* (%)	217 (43.9%)	120 (49.2%)	97 (38.8%)	
BMI, mean (range)	26.62 (15.6–45.6)	26.06 (15.6–45.6)	27.23 (17.3–42.5)	**0.003**
Past smoking, *n* (%)	99 (20%)	44 (18%)	55 (22%)	0.27
Current smoking, *n* (%)	92 (18.6%)	44 (18%)	48 (19.2%)	0.74
ASA score				
Median (IQR)	3 (1)	3 (1)	3 (1)	0.77
1, *n* (%)	19 (3.8%)	11 (4.5%)	8 (3.2%)	
2, *n* (%)	149 (30.2%)	74 (30.3%)	75 (30%)	
3, *n* (%)	249 (50.4%)	122 (50%)	127 (50.8%)	
4, *n* (%)	14 (2.8%)	7 (2.9%)	7 (2.8%)	
5, *n* (%)	1 (0.2%)	1 (0.4%)	0 (0%)	
Missing, *n* (%)	62 (12.6%)	29 (11.9%)	33 (13.2%)	
Comorbidities prevalence				
Any, *n* (%)	391 (79.1%)	193 (79.1%)	198 (79.2%)	0.98
CVA/TIA, *n* (%)	17 (3.4%)	9 (3.7%)	8 (3.2%)	0.77
Asthma, *n* (%)	46 (9.3%)	21 (8.6%)	25 (10%)	0.59
IHD, *n* (%)	67 (13.6%)	34 (13.9%)	33 (13.2%)	0.81
Arrhythmia, *n* (%)	28 (5.7%)	14 (5.7%)	14 (5.6%)	0.95
DM, *n* (%)	112 (22.7%)	48 (19.7%)	64 (25.6%)	0.12
CRF, *n* (%)	15 (3%)	7 (2.9%)	8 (3.2%)	0.83
HTN, *n* (%)	217 (43.9%)	111 (45.5%)	106 (42.4%)	0.49
Dyslipidemia, *n* (%)	172 (34.8%)	81 (33.2%)	91 (36.4%)	0.46
Family hx of CRC				
1st degree, *n* (%)	77 (15.6%)	30 (12.3%)	47 (18.8%)	**0.046**
Non-1st degree, *n* (%)	39 (7.9%)	18 (7.4%)	21 (8.4%)	0.67
Abdominal Surgery hx, *n* (%)	295 (59.7%)	147 (60.2%)	148 (59.2%)	0.81
Bowel surgery hx, *n* (%)	55 (11.1%)	24 (9.8%)	31 (12.4%)	0.37

OT—operative time, BMI—body mass index, ASA—American Society of Anesthesiologists, CVA—cerebrovascular accident, TIA—transient ischemic attack, IHD—ischemic heart disease, CRF—chronic renal failure, DM—diabetes mellitus, HTN—hypertension, CRC—colorectal cancer. bold for *p* < 0.05.

**Table 2:** Preoperative characteristics.

Variable	All Cohort (*n* = 494)	Short OT (*n* = 244)	Long OT (*n* = 250)	*p* Value
Preop CEA, median (IQR)	2.0 (2.58)	1.8 (2.55)	2.0 (2.5)	0.29
Preop CA 19-9, median (IQR)	9.3 (13.2)	9.8 (15.26)	9.0 (12.8)	0.9
Symptoms present, *n* (%)	396 (80.2%)	190 (77.9%)	206 (82.4%)	0.21
Abdominal pain, *n* (%)	88 (17.8%)	40 (16.4%)	48 (19.2%)	0.42
Anemia, *n* (%)	44 (8.9)	22 (9%)	22 (8.8%)	0.93
Weight loss, *n* (%)	101 (20.4%)	42 (17.2%)	59 (23.6%)	0.08
BM change, *n* (%)	205 (41.5%)	96 (39.3%)	109 (43.6%)	0.34
LGIB, *n* (%)	287 (58.1%)	138 (56.6%)	149 (59.6%)	0.49
Tumor location				**<0.001**
Lower rectum, *n* (%)	57 (11.5%)	19 (7.8%)	38 (15.2%)	
Mid rectum, *n* (%)	159 (32.2%)	59 (24.2%)	100 (40%)	
Upper rectum, *n* (%)	278 (56.3%)	166 (68%)	112 (44.8%)	
Distance from AV (cm), mean (range)	9.99 (0–15)	10.94 (0–15)	9.04 (0–15)	**<0.001**
Neoadjuvant therapy, any	248 (50.2%)	100 (41%)	148 (59.2%)	**<0.001**
Chemoradiation, Long, *n* (% *)	216 (87.1%)	86 (86%)	130 (87.8%)	**<0.001**
Chemoradiation, Short, *n* (% *)	30 (12.1%)	14 (14%)	16 (10.8%)	0.76
TNT, *n* (% *)	19 (7.7%)	5 (5%)	14 (9.5%)	**0.04**
Clinical stage				**0.049**
Stage 1, *n* (%)	114 (23.1%)	54 (22.1%)	60 (24%)	
Stage 2, *n* (%)	97 (19.6%)	49 (20.1%)	48 (19.2%)	
Stage 3, *n* (%)	243 (49.2%)	113 (46.3%)	130 (52%)	
missing, *n* (%)	40 (8.1%)	28 (11.5%)	12 (4.8%)	
T stage				0.052
T1, *n* (%)	41 (8.3%)	23 (9.4%)	18 (7.2%)	
T2, *n* (%)	98 (19.8%)	47 (19.3%)	51 (20.4%)	
T3, *n* (%)	280 (56.7%)	132 (54.1%)	148 (59.2%)	
T4, *n* (%)	35 (7.1%)	14 (5.7%)	21 (8.4%)	
missing, *n* (%)	40 (8.1%)	28 (11.5%)	12 (4.8%)	
N stage				**0.033**
N0, *n* (%)	221 (44.7%)	109 (44.7%)	112 (44.8%)	
N1, *n* (%)	175 (35.4%)	85 (34.8%)	90 (36%)	
N2, *n* (%)	68 (13.8%)	28 (11.5%)	40 (16%)	
missing, *n* (%)	30 (6.1%)	22 (9%)	22 (5.3%)	
Rectal wall tumor location				0.56
anterior, *n* (%)	101 (20.4%)	47 (19.3%)	54 (21.6%)	
Posterior, *n* (%)	99 (20%)	42 (17.2%)	57 (22.8%)	
Circumferential, *n* (%)	73 (14.8%)	37 (15.2%)	36 (14.4%)	
Preop Albumin, mean (range)	4.06 (2.4–5.1)	4.09 (2.5–5.1)	4.04 (2.4–4.8)	0.16
Preop Hgb, mean (range)	12.68 (7.76–17.2)	12.7 (8.7–17.2)	12.7 (7.76–16.7)	0.98
Preop Creatinine, median (IQR)	0.84 (0.3)	0.84 (0.29)	0.82 (0.3)	0.43

* % out of the patients receiving neoadjuvant therapy; OT—operative time, CEA—carcinoembryonic antigen, BM—bowel movements, LGIB—lower gastrointestinal bleeding, AV—anal verge, TNT—total neoadjuvant therapy, Hgb—Hemoglobin. bold for *p* < 0.05.

**Table 3:** Operative and perioperative details.

Variable	All Cohort (*n* = 494)	Short OT (*n* = 244)	Long OT (*n* = 250)	*p* Value
Surgical approach				0.67
Open, *n* (%)	130 (26.3%)	63 (25.8%)	67 (26.8%)	
Laparoscopic, *n* (%)	262 (53%)	134 (54.9%)	128 (51.2%)	
Robotic, *n* (%)	102 (20.6%)	47 (19.3%)	55 (22%)	
Conversion, *n* (% *)	69 (19.0%)	27 (14.9%)	42 (22.9%)	0.051
Skin incision				**0.029**
Midline, *n* (%)	194 (39.3%)	89 (36.5%)	105 (42%)	
Pfanensteil, *n* (%)	227 (46%)	126 (51.6%)	101 (40.4%)	
Other, *n* (%)	73 (14.8%)	29 (11.9%)	44 (17.6%)	
Surgery type				
Anterior resection, *n* (%)	166 (33.6%)	109 (44.7%)	57 (22.8%)	**<0.001**
Low anterior resection, *n* (%)	294 (59.5%)	125 (51.2%)	169 (67.6%)	**<0.001**
Abdominoperineal resection, *n* (%)	32 (6.5%)	10 (4.1%)	22 (8.8%)	**0.03**
Extended resections, *n* (%)	78 (15.8%)	24 (9.8%)	54 (21.6%)	**<0.001**
Additional Anastomosis, *n* (%)	17 (3.4%)	2 (0.8%)	15 (6%)	**0.002**
BSO, *n* (%)	37 (7.5%)	12 (4.9%)	25 (10%)	**0.03**
Hysterectomy, *n* (%)	9 (1.8%)	0 (0%)	9 (3.6%)	**0.004**
Hernia, *n* (%)	6 (1.2%)	4 (1.6%)	2 (0.8%)	0.45
Cholecystectomy, *n* (%)	3 (0.6%)	0 (0%)	3 (1.2%)	0.25
Intra-op complications, *n* (%)	79 (16%)	25 (10.2%)	54 (21.6%)	**<0.001**
Bleeding, *n* (%)	25 (5.1%)	8 (3.3%)	17 (6.8%)	0.07
Ureter injury, *n* (%)	4 (0.8%)	1 (0.4%)	3 (1.2%)	0.62
Enterotomy, *n* (%)	21 (4.3%)	5 (2%)	16 (6.4%)	**0.02**
Splenic injury, *n* (%)	7 (1.4%)	3 (1.2%)	4 (1.6%)	0.99
Vaginal injury, *n* (%)	8 (1.6%)	2 (0.8%)	6 (2.4%)	0.29
Anastomotic disruption, *n* (%)	8 (1.6%)	2 (0.8%)	6 (2.4%)	0.29
Stoma diversion, *n* (%)	305 (61.8%)	112 (45.9%)	193 (77.2%)	**<0.001**
Stoma reversal, n (%/diverted)	210 (68.9%)	72 (64.3%)	138 (71.5%)	0.19
Surgery duration (minutes), median (IQR)	270 (155)	201 (125)	340 (110)	**<0.001**
LOS (days), median (mode, IQR)	8 (7, 4)	8 (7, 4)	8 (7, 4)	**<0.001**
Post-operative complication, *n* (%)	331 (67%)	148 (60.7%)	183 (73.2%)	**0.003**
SSI, *n* (%)	71 (14.4%)	30 (12.3%)	41 (16.4%)	0.19
Abscess, *n* (%)	36 (7.3%)	11 (4.5%)	25 (10%)	**0.02**
Ileus/SBO, *n* (%)	108 (21.9%)	51 (20.9%)	57 (22.8%)	0.61
Anastomotic leak, *n* (%)	43 (8.7%)	16 (6.6%)	27 (10.8%)	0.09
Bleeding, *n* (%)	69 (14%)	25 (10.2%)	44 (17.6%)	**0.02**
Pneumonia, *n* (%)	11 (2.2%)	5 (2%)	6 (2.4%)	0.79
UTI, *n* (%)	31 (6.3%)	12 (4.9%)	19 (7.6%)	0.22
DVT, *n* (%)	3 (0.6%)	2 (0.8%)	1 (0.4%)	0.62
ACS, *n* (%)	17 (3.4%)	8 (3.3%)	9 (3.6%)	0.85
Wound dehiscence, *n* (%)	9 (1.8%)	3 (1.2%)	6 (2.4%)	0.50
Electrolyte, *n* (%)	181 (36.6%)	83 (34%)	98 (39.2%)	0.23
Urinary retention, *n* (%)	38 (7.7%)	16 (6.6%)	22 (8.8%)	0.35
Clavien Dindo score, median (IQR)	1 (2)	1 (2)	1 (2)	**<0.001**
Severe complications, *n* (%)	81 (16.4%)	32 (13.1%)	49 (19.6%)	0.052
30-day readmission, *n* (%)	88 (17.8%)	42 (17.2%)	46 (18.4%)	0.73
30-day mortality, *n* (%)	6 (1.2%)	2 (0.8%)	4 (1.6%)	0.69

* % out of the patients undergoing minimally invasive surgery; OT—operative time, BSO—bilateral salpingo-oophorectomy, SSI—surgical site infection, SBO—small bowel obstruction, UTI—urinary tract infection, DVT—deep vein thrombosis, ACS—acute coronary syndrome, LOS—length of stay. bold for *p* < 0.05.

**Table 4:** Pathological results.

Variable	All Cohort (*n* = 494)	Short OT (*n* = 244)	Long OT (*n* = 250)	*p* Value
Complete pathological response, *n* (% *)	54 (21.8%)	26 (26%)	28 (18.9%)	0.19
Pathological tumor size (cm), mean (range)	3.06 (0–11)	3.25 (0.2–10.2)	2.88 (0–11)	**0.025**
Tumor differentiation				0.76
Well differentiated, *n* (%)	132 (26.7%)	66 (27%)	66 (26.4%)	
Moderately differentiated, *n* (%)	210 (42.5%)	107 (43.9%)	103 (41.2%)	
Poorly differentiated, *n* (%)	18 (3.6%)	7 (2.9%)	11 (4.4%)	
Pathological staging				0.26
0, *n* (%)	54 (10.9%)	26 (10.7%)	28 (11.2%)	
I, *n* (%)	168 (34%)	79 (32.4%)	89 (35.6%)	
II, *n* (%)	116 (23.5%)	60 (24.6%)	56 (22.4%)	
III, *n* (%)	147 (29.8%)	74 (30.3%)	73 (29.2%)	
IV, *n* (%)	5 (1%)	1 (0.4%)	4 (1.6%)	
Pathological T staging				0.44
0, *n* (%)	57 (11.5%)	27 (11.1%)	30 (12%)	
1, *n* (%)	61 (12.3%)	25 (10.2%)	36 (14.4%)	
2, *n* (%)	135 (27.3%)	68 (27.2%)	67 (26.8%)	
3, *n* (%)	227 (46.0%)	117 (48%)	110 (44%)	
4, *n* (%)	10 (2%)	3 (1.2%)	7 (2.8%)	
Pathological N staging				0.93
0, *n* (%)	338 (68.4%)	164 (67.2%)	174 (69.6%)	
1, *n* (%)	122 (24.7%)	61 (25%)	61 (24.4%)	
2, *n* (%)	28 (5.7%)	13 (5.3%)	15 (6%)	
LVI, *n* (%)	38 (7.7%)	19 (7.8%)	19 (7.6%)	0.94
Perineural invasion, *n* (%)	39 (7.9%)	18 (7.4%)	21 (8.4%)	0.67
Number of nodes harvested, median (IQR)	14 (9)	14 (9.75)	14 (8)	0.99
Distal margin involved (cm), *n* (%)	5 (1%)	3 (1.2%)	2 (0.8%)	0.68
Distance from distal margin, mean (range)	2.67 (0.14–9)	3.06 (0.2–9)	2.29 (0.14–6)	**0.008**
Radial margin involved, *n* (%)	5 (1%)	2 (0.8%)	3 (1.2%)	0.99
Signet ring, *n* (%)	11 (2.2%)	5 (2%)	6 (2.4%)	0.72
Mucin present, *n* (%)	85 (17.2%)	32 (13.1%)	53 (21.2%)	**0.017**

* Out of patients who received neoadjuvant treatment, OT—operative time, LVI—lymphovascular invasion. bold for *p* < 0.05.

**Table 5:** Cox regression analysis for associations with recurrence-free survival.

Variable	Univariate			Multivariate		
	HR	95% CI	*p*	HR	95% CI	*p*
Age	1.01	0.99–1.02	0.49			0.54
BMI	0.98	0.95–1.02	0.42			0.55
Distance from AV	0.95	0.92–0.99	**0.023**	0.95	0.91–0.99	**0.035**
Preop CEA	1.01	1.01–1.02	**<0.001**			0.2
Surgery duration quartile	1.3	1.10–1.54	**0.002**	1.37	1.12–1.69	**0.002**
Severe complication	1.2	0.76–1.9	0.43			0.88
Neoadjuvant treatment	0.85	0.61–1.19	0.35			0.80
Pathological tumor size	1.09	0.98–1.20	0.11			0.055
Pathological T stage	1.94	1.54–2.46	**<0.001**	1.82	1.28–2.58	**<0.001**
Pathological stage	1.33	1.22–1.46	**<0.001**	1.19	1.01–1.42	**0.04**
Margin involvement (distal/radial)	4.99	2.53–9.85	**<0.001**	3.4	1.54–7.51	**0.002**
Pathological mucinous	0.63	0.42–0.94	**0.024**			0.95

BMI—body mass index, AV—anal verge, CEA—carcinoembryonic antigen. bold for *p* < 0.05.

**Table 6:** Regression analysis for predictors of longer surgery time.

Variable	Univariate			Multivariate		
	OR	95% CI	*p*	AOR	95% CI	*p*
Age	1.00	0.99–1.02	0.57			0.53
Male gender	1.52	1.06–2.17	**0.02**			0.059
BMI	1.06	1.02–1.10	**0.005**	1.07	1.02–1.11	**0.002**
Distance from AV	0.89	0.85–0.93	**<0.001**	0.89	0.85–0.93	**<0.001**
Preop long chemoradiation	1.99	1.39–2.86	**<0.001**			0.35

AOR—adjusted odds ratio, BMI—body mass index, AV—anal verge. bold for *p* < 0.05.

## Data Availability

Due to ethical considerations and privacy regulations, study datasets can be provided by the corresponding author upon reasonable request.

## Supplementary Materials

The following supporting information can be downloaded at: https://www.mdpi.com/article/10.3390/jcm15218473/s1, Table S1. Case distribution and median operative times stratified by primary surgeon and surgical approach.

## Abbreviations

The following abbreviations are used in this manuscript:

OT	Operative time
pCR	Pathological complete response
LARC	Locally advanced rectal cancer
LCCRT	Long-course chemoradiotherapy
SCRT	Short-course radiotherapy
TME	Total Mesorectal Excision
CCR	Clinical complete response
TNT	Total neoadjuvant therapy
APR	Abdominoperineal Resection
BMI	Body Mass Index
ASA	American Society of Anesthesiologists
LOS	Length Of Stay
RFS	Recurrence-Free Survival
OS	Overall Survival
CRC	ColoRectal Cancer
TIA	Transient Ischemic Attack
CVA	Cerebral Vascular Accident
COPD	Chronic Obstructive Pulmonary Disease
IHD	Ischemic Heart Disease
CHF	Congestive Heart Failure
DM	Diabetes Melitus
CKD	Chronic Kidney Disease
HTN	Hypertension
EMVI	Extramural Vascular Invasion
BSO	Bilateral Salpingo-oophorectomy
SSI	Surgical Site Infection
SBO	Small Bowel Obstruction
UTI	Urinary Tract Infection
DVT	Deep Vein Thrombosis
DLI	Diverting Loop Ileostomy
AV	Anal Verge
