# Supplementary material for: Predictors and Prognostic Implications of Prolonged Operative Time in Rectal Cancer Surgery: An Analysis Adjusted for Surgeon and Surgical Approach
Source: J Clin Med. 2026 Oct 30;15(21):8473. doi: 10.3390/jcm15218473 (PMC13763774; doi:10.3390/jcm15218473)
Supplement: Supplementary file 1 [file jcm-15-08473-s001.zip › jcm-4546787-supplementary.pdf]

Table S1. Case distribution and median operative times stratified by primary surgeon and surgical approach (N = 494).

Surgeon ID	Surgical Approach	Case Volume (n)	Median Duration (min)	Range (min)
Other / Low Volume	Open	23	230.0	100 – 600
Other / Low Volume	Laparoscopic	10	182.5	75 – 360
Other / Low Volume	Robotic	1	590.0	590 – 590
Other / Low Volume	***Combined Minimally Invasive***	*11*	*190.0*	*75 – 590*
Surgeon A	Laparoscopic	14	210.0	130 – 637
Surgeon A	***Combined Minimally Invasive***	*14*	*210.0*	*130 – 637*
Surgeon B	Open	8	241.0	235 – 380
Surgeon B	Laparoscopic	49	305.0	86 – 540
Surgeon B	Robotic	88	315.0	46 – 525
Surgeon B	***Combined Minimally Invasive***	*137*	*310.0*	*46 – 540*
Surgeon C	Open	10	234.5	130 – 960
Surgeon C	Laparoscopic	52	300.0	125 – 583
Surgeon C	Robotic	1	318.0	318 – 318
Surgeon C	***Combined Minimally Invasive***	*53*	*300.0*	*125 – 583*
Surgeon D	Open	50	142.5	80 – 390
Surgeon D	***Combined Minimally Invasive***	*0*	*—*	*—*
Surgeon E	Laparoscopic	18	147.5	85 – 285
Surgeon E	***Combined Minimally Invasive***	*18*	*147.5*	*85 – 285*
Surgeon F	Open	7	250.0	100 – 300
Surgeon F	Laparoscopic	39	335.0	170 – 465
Surgeon F	***Combined Minimally Invasive***	*39*	*335.0*	*170 – 465*
Surgeon G	Open	11	195.0	140 – 370
Surgeon G	Laparoscopic	24	240.0	90 – 494
Surgeon G	***Combined Minimally Invasive***	*24*	*240.0*	*90 – 494*
Surgeon H	Laparoscopic	10	140.0	75 – 550
Surgeon H	Robotic	1	250.0	250 – 250
Surgeon H	***Combined Minimally Invasive***	*11*	*160.0*	*75 – 550*
Surgeon I	Open	21	225.0	100 – 475
Surgeon I	Laparoscopic	36	240.0	120 – 420

Surgeon I	Robotic	3	280.0	260 – 310
Surgeon I	***Combined Minimally Invasive***	*39*	*260.0*	*120 – 420*
Surgeon J	Laparoscopic	10	354.0	200 – 512
Surgeon J	Robotic	8	342.5	270 – 560
Surgeon J	***Combined Minimally Invasive***	*18*	*354.0*	*200 – 560*
**Total Cohort**	**All Approaches**	**494**	**270.0**	**46 – 960**

* Note: Surgeons are anonymized as Surgeons A–J. Low-volume surgeons and low-frequency individual approach strata were merged into the 'Other / Low Volume' category. Minimally invasive procedures (Laparoscopic and Robotic) were combined into a unified stratum for primary median standardization to prevent small-stratum instability in low-volume robotic cases. Cases with durations equal to or below the calculated stratum median were assigned to the Short OT group (n = 244), and those exceeding the median to the Long OT group (n = 250).
